# Coincidence of Incomplete Pentalogy of Cantrell and Meningomyelocele in a Dizygotic Twin Pregnancy

**DOI:** 10.1155/2015/629561

**Published:** 2015-09-01

**Authors:** Hakan Timur, Aytekin Tokmak, Hatice Bayram, Esra Şükran Çakar, Nuri Danışman

**Affiliations:** ^1^Department of Obstetrics and Gynecology, Zekai Tahir Burak Women's Health Research and Education Hospital, 06230 Ankara, Turkey; ^2^Department of Pathology, Zekai Tahir Burak Women's Health Research and Education Hospital, 06230 Ankara, Turkey; ^3^Department of Medical Genetics, Zekai Tahir Burak Women's Health Research and Education Hospital, 06230 Ankara, Turkey

## Abstract

Pentalogy of Cantrell is an extremely rare and lethal syndrome. Ectopia cordis is frequently found in fetuses with POC but not required for incomplete forms. Likewise, meningomyelocele is a relatively uncommon neural tube defect affecting central nervous system and associated with neurological problems. Herein, we presented a woman with dizygotic twin pregnancy having coincidence of incomplete POC and MMC in each individual fetus, which has never been reported previously.

## 1. Introduction

Pentalogy of Cantrell (POC) is an extremely rare and lethal syndrome originally described by Cantrell in 1958, with an incidence estimated at around 6 per million live births [1]. The classical pentad for this disorder includes the following 5 main features; omphalocele, ectopia cordis (abnormal location of heart), diaphragmatic defect, pericardial defect or sternal cleft, and intracardiac defects [2]. POC occurs with varying degrees of severity, and only few patients show all five of the defects.

Meningomyelocele (MMC) is the most common neural tube defect, with an incidence of about 1 per one thousand live births [3]. It is characterized by a cleft in the vertebral column, in which meninges and spinal cord are exposed directly to the amniotic fluid. It may occur anywhere in the spine, from the cervical to the sacral region. The severity of neurologic dysfunction in newborns with MMC varies according to the localization and dimensions of the lesion, and the morphological appearance varies considerably depending on the developmental stage at which the embryo or fetus is examined [4].

It is well known that monozygotic twinning is associated with increased risk of structural birth defects [5]. However, coincidence of these defects in dizygotic twins is extremely rare. Herein, we presented a woman with dizygotic twin pregnancy having coincidence of POC and MMC in each individual fetus, which has never been reported previously.

## 2. Case Report

A 32-year-old, gravida 3 para 1, woman presented to our perinatology outpatient clinic with a dichorionic twin pregnancy at the 18th week of gestation. She was referred to our clinic to be investigated for the cause of elevated serum levels of alpha-fetoprotein. Her past obstetric history included an early spontaneous abortion and a twin birth. Because of anovulation, she had received clomiphene citrate in her previous pregnancy and had given birth to twins by cesarean section. One of the dizygotic twins was healthy and the other had spastic paraparesis. The parents were nonconsanguineous and healthy. The patient wishing to become pregnant again had been able to conceive after treatment with clomiphene citrate (CC) in the current pregnancy. Her husband had normal semen parameters; therefore, insemination was performed. She was living in low socioeconomic conditions, was nonsmoker, and did not receive any vitamin supplementation before this pregnancy. She was a housewife with no history of substance abuse, drug use, or potential teratogen exposure. She had only received oral micronized progesterone because of threatened miscarriage in the first trimester. Her past medical history and physical examination were also unremarkable.

**Figure 1:**
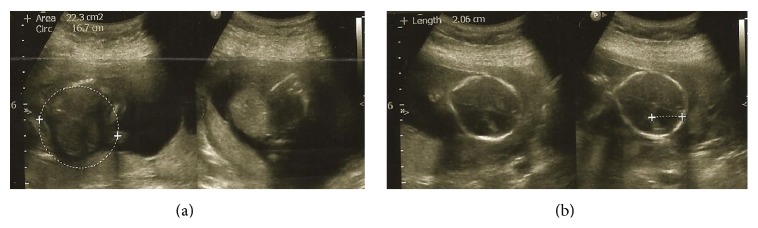
The image of the prenatal ultrasound scan. (a) The omphalocele sac (arrowhead) containing the liver and bowels. (b) The lemon sign (arrowhead).

An ultrasound scan was performed using a General Electric Logiq A5 (Milwaukee, USA) with an abdominal transducer (3.5 Hz) and showed a dichorionic-diamniotic twin pregnancy. Fetal cardiac activity was present in both fetuses. The measurements of the femur length and biparietal diameter were compatible with 17 and 18 weeks of gestation in the first and second fetuses, respectively. The detailed sonographic scan of the first fetus revealed a large ventricular septal defect and an intact omphalocele containing the liver and bowel loops (Figure 1(a)). The diagnosis of POC was suspected in the first twin. The lemon sign was present in the second twin, and the diagnosis of lumbosacral MMC was made (Figure 1(b)). Based on the findings of POC in the first fetus and MMC in the other by prenatal ultrasound screening, the parents were informed about the anomalies. They were told that repeated surgical interventions with poor prognosis may be required for both fetuses, and termination of the pregnancy was recommended. She and her family elected to terminate the pregnancy.

**Figure 2:**
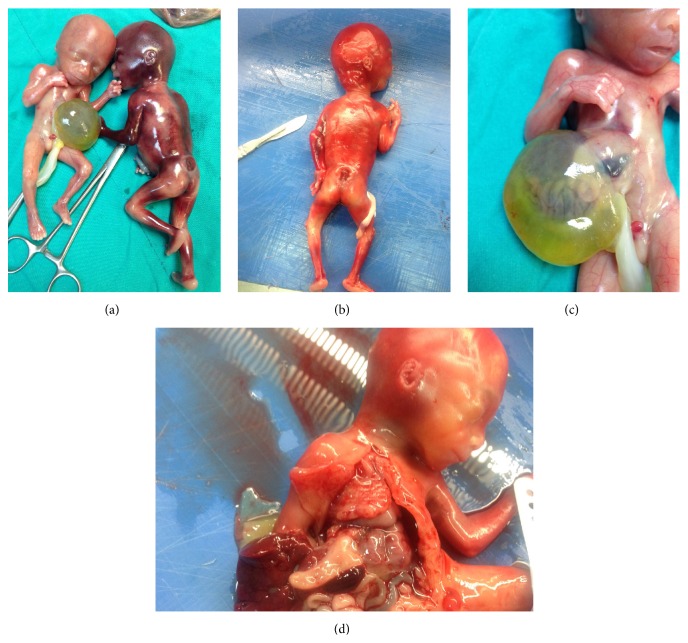
Postabortal appearance of both fetuses. (a) The omphalocele sac. (b) The lumbosacral meningomyelocele. (c) The bifid sternum. (d) The absence of diaphragm.

The diagnosis was confirmed on the postmortem examination and there were no karyotype anomalies; 46,XY was observed in both fetuses. In the radiographic evaluation, bone structure showed no gross pathology. At the autopsy of the first fetus, absence of the diaphragm with a sternal cleft (bifid sternum), a large omphalocele in which the intestines, liver, accessory spleen, and stomach remained outside the abdomen in the sac, and an intraventricular saptal defect were reported (Figure 2). The second twin was macroscopically normal with the exception of sacral MMC. An autopsy was not performed on him.

## 3. Discussion

Congenital malformations are important causes of perinatal morbidity and mortality. Although many syndromes associated with these malformations have been reported in the literature, POC is one of them, which is extremely rare. Approximately, 120 cases have been reported around the world until now. It is a syndrome with high mortality rate and the survival rate is lower than 40% [6]. The etiopathogenesis of this disorder still remains unclear, but the most accepted reason is developmental failure of a segment of the lateral mesoderm in early embryonic life [2]. POC may display different phenotypic features, and patients with POC may be classified into three groups as suggested by Toyama [7]. A diagnosis of complete POC requires the five criteria, but for diagnoses of incomplete variants, three or four features are required. The incomplete variants of POC include probable diagnosis (four defects are present, including intracardiac and ventral wall abnormalities) and incomplete form (incomplete expression and various combinations of defects including a sternal abnormality) [7]. Few cases of incomplete variants of POC are reported in the literature.

Various intracardiac anomalies have been described in the POC patients including ventricular septal defects (VSD) (100%), atrial septal defects (ASD) (52%), tetralogy of Fallot (TOF) (20%), and pulmonary stenosis (33%) [2]. Otherwise, de Rubens Figueroa et al. [8] showed that double outlet right ventricle (DORV) was the most common heart disease among the newborns with diagnosed POC. Ectopia cordis is often found in fetuses with POC but not required for incomplete forms. Our case is one of several cases without ectopia cordis.

Abdominal wall defects encountered in POC are omphalocele, epigastric hernia, umbilical hernia, diastasis recti, and combined defects. Sternal malformations include bifid sternum, absent xiphoid, short sternum, and defective formation of the lower two-thirds [9]. Differential diagnosis of midline defects includes POC, limp body walk complex, body stalk anomaly, and amniotic band syndrome [10]. Also, craniofacial (e.g., cleft lip and palate) and central nervous system (e.g., hydrocephalus) anomalies, limb defects (e.g., clubfoot, tibia, and radius agenesis), and intra-abdominal anomalies (e.g., polysplenia and gallbladder agenesis) are the other associated anomalies. Omphalocele, ventricular septal defect, bifid sternum and absence of diaphragm were noted in the reported case as well.

There is male dominance with a male to female ratio of 2.7 : 1 in POC syndrome. Although most cases of POC are seemingly sporadic, some authors have suggested that there could be a genetic component. This syndrome was reported in a monozygotic twin pregnancy with Trizomi 18 [11]. Also in the literature, there is a report of 2 consecutive born brothers with POC suggesting that the disease may be hereditary [12]. Our twins were also males, but their karyotypes were normal and there is no family history of POC.

Intrauterine diagnosis of this pentalogy is impossible before the 12th week of gestation, because of herniation of bowel out of abdomen is a normal event in fetal development at this time, but after that ultrasonography is a useful method even in the first trimester [13]. Both 2-dimensional ultrasonography and 3-dimensional obstetric ultrasonography are recommended, but 3D ultrasonography is not necessary in first trimester. Other diagnostic methods including computerized tomography and magnetic resonance can be used for confirmation. We were unable to diagnose in the first trimester, because late referral caused delay in the diagnosis. However, 2-dimensional ultrasonography was sufficient for the initial diagnosis in this case. And a definitive diagnosis of POC was made after autopsy.

The optimal management strategies and prognostic indices for neonates remain to be established. Generally, most of the fetuses associated with ectopia cordis indicate poor prognosis, whereas ectopia cordis in partial association with incomplete presentation of POC is likely more favorable [14]. Multiple corrective surgical interventions may also be required in these cases.

Meningomyelocele is a relatively common disorder. It is a neural tube defect (NTD) that results from a failure of closure of the neural tube during the 4th week of embryogenesis. Etiology is variable, involving both genetic and environmental factors, for instance, lack of folic acid supplementation during embryonary neurulation. Maternal obesity and gestational diabetes may also increase the risk of NTDs [15]. The genetic contribution to malformation of neural tube is suggested by the predominance of its occurrence among families with a history of neural tube defect. A prenatal diagnosis by ultrasound scan is sometimes difficult, and it usually reveals only the hydrocephalus and distortions associated with hydrocephalus. Our case was a woman of normal weight without history of diabetes. And she had no previous history of pregnancy complicated by NTD.

There are limited case reports about the combination of POC with neural tube defects [16]. As far as we know, this is the first report of coincidence of POC and MMC in a dizygotic twin pregnancy. Our patient conceived with CC treatment. Although some earlier studies suggested an association between the use of CC and certain congenital anomalies, there is no evidence that CC treatment increases the risk of birth defects [17]. In the present case, lower socioeconomic status and lack of folic acid supplementation during pregnancy may be the cause, especially for the MMC.

In conclusion, prenatal diagnosis is possible and early counseling should be made to make differential diagnosis. When the POC is diagnosed, a multidisciplinary team including an obstetrician, a neonatologist, a pediatric cardiologist, a pediatric surgeon, and a genetics specialist should inform the families about the prognosis of the disease. And the option of termination of pregnancy should be available for these patients.
